# Predictive value of inflammatory burden index for new-onset atrial fibrillation in STEMI patients

**DOI:** 10.3389/fcvm.2025.1599152

**Published:** 2025-09-16

**Authors:** Kun Liu, Zhiwen Tao, Gonghao Li, Mingzhu Li, Jiayu Yin, Lei Zhou

**Affiliations:** ^1^ Department of Cardiology, First Affiliated Hospital of Nanjing Medical University, Nanjing, Jiangsu, China; ^2^ Department of Cardiology, The First People’s Hospital of Lianyungang, Lianyungang, Jiangsu, China; ^3^ Department of Cardiology, The Second Affiliated Hospital of Soochow University, Suzhou, Jiangsu, China

**Keywords:** inflammation response, inflammatory burden index, atrial fibrillation, ST-segment elevation myocardial infarction, risk stratification

## Abstract

**Background:**

The inflammatory burden index (IBI) is a novel and useful inflammatory marker. However, the association between IBI and new-onset atrial fibrillation (*N*OAF) in patients with ST-segment elevation myocardial infarction (STEMI) remains unclear. This study focuses on exploring the predictive ability of IBI for NOAF after percutaneous coronary intervention (PCI) in STEMI patients.

**Materials and methods:**

This study is a single-center retrospective observational study. Patients diagnosed with STEMI and undergoing primary PCI between October 2022 and February 2025 were continuously enrolled. All enrolled patients received continuous electrocardiogram (ECG) monitoring (>72 h) and were grouped according to whether NOAF occurred during hospitalization. Logistic regression analysis was used to identify potential risk factors for NOAF. Meanwhile, restricted cubic spline (RCS) analysis was employed to thoroughly investigate the possible dose-response relationship between IBI and NOAF.

**Results:**

A total of 696 STEMI patients were finally included in this study. The incidence of NOAF during hospitalization was 62/696 (8.9%). After adjusting for potential confounding factors, the results of multivariate logistic regression analysis showed that left ventricular ejection fraction (OR = 0.928, 95% CI: 0.895–0.962), age (OR = 1.048, 95% CI: 1.022–1.075), and IBI (OR = 1.007, 95% CI: 1.003–1.011) were independent factors for NOAF in STEMI patients (*P* < 0.05). RCS results suggested that there was a non-linear dose-response relationship between IBI and NOAF. After integrating IBI, the ability of the new model to predict NOAF was significantly improved (*N*RI = 0.617, 95% CI: 0.360–0.873, *P* < 0.01; IDI = 0.026, 95% CI: 0.007–0.046, *P* = 0.008).

**Conclusions:**

Elevated IBI is an independent risk factor for NOAF after PCI in STEMI patients. Integrating IBI can improve the risk stratification for NOAF in STEMI patients.

## Background

ST-segment elevation myocardial infarction (STEMI) is one of the major diseases threatening human health (1). Percutaneous coronary intervention (PCI) has currently become an important treatment modality for STEMI, which can significantly improve the prognosis of patients. However, STEMI patients still face numerous complications after PCI. Among them, new-onset atrial fibrillation (NOAF) is one of the most common complications (2, 3). NOAF can significantly increase the risk of adverse events such as thrombus formation, heart failure, and sudden cardiac death (3). More problematically, when atrial fibrillation (AF) coexists with STEMI, the condition becomes more complicated, and there are many dilemmas in the treatment process (4). Therefore, exploring the risk factors of NOAF and establishing a precise and effective risk stratification system are of great significance for improving the prognosis of STEMI patients.

Inflammation plays an important role in the occurrence and development of STEMI and AF. During the onset of STEMI, from the unstable rupture of coronary atherosclerotic plaques to myocardial ischemia-reperfusion injury, the inflammatory response persists throughout and plays a crucial role (5). Similarly, in the pathogenesis of AF, inflammation is considered one of the important factors triggering and maintaining atrial remodeling (6). In recent years, the inflammatory burden index (IBI), as an emerging inflammatory marker, has been proven to be closely associated with various diseases (7–9). However, the relationship between IBI and NOAF in STEMI patients remains unclear. This study aims to explore the relationship between IBI and NOAF in STEMI patients, with the hope of providing valuable references for clinical practice.

## Material and methods

### Study population

**Figure 1:**
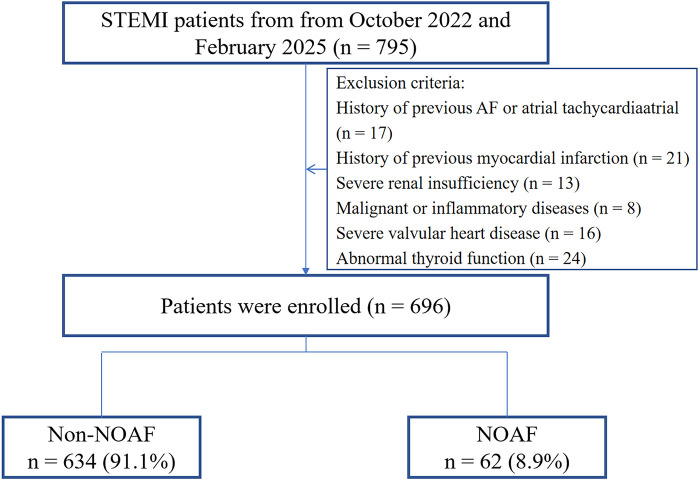
Study flowchart. STEMI ST-segment elevation myocardial infarction; NOAF, new-onset atrial fibrillation.

This single-center, retrospective observational study included consecutive patients diagnosed with STEMI (10) at the First People's Hospital of Lianyungang between October 2022 and February 2025. The study protocol was approved by the institutional review board of the First People's Hospital of Lianyungang (KY−20250213001-01). Given the no risk to participants, the requirement for written informed consent was waived. Inclusion criteria were: successful primary PCI within 12 h of symptom onset with post-procedural TIMI grade 3 flow; continuous electrocardiographic (ECG) monitoring for (>72 h) during hospitalization; and availability of complete clinical data. Exclusion criteria included: age <18 years; prior AF or atrial tachycardia; prior myocardial infarction; severe renal insufficiency (GFR < 30 ml/min/1.73 m^2^); malignancy or inflammatory diseases (any acute or chronic inflammatory condition within 3 months prior to enrollment that could affect inflammatory biomarkers, including autoimmune diseases, chronic inflammatory conditions, and moderate-to-severe infections); severe valvular heart disease; and thyroid dysfunction. In total, 696 patients were included in the analysis, of whom 62 developed NOAF (Figure 1).

### Clinical data collection

Baseline clinical data were collected for all patients, including sex, age, body mass index (BMI), smoking status, and medical history. Lymphocyte count, neutrophil count, and C-reactive protein (CRP) level were measured before PCI. The neutrophil-to-lymphocyte ratio (NLR) was defined as the ratio of neutrophil to lymphocyte counts, and IBI was defined as the product of CRP and NLR (7–9). During hospitalization, fasting venous blood samples were obtained for laboratory testing, including blood lipids and glucose. The peak values of Troponin T (TnI) and N-terminal pro–B-type natriuretic peptide (NT-proBNP) were recorded. AF was defined as an arrhythmia documented on a single-lead ECG lasting (≥30 s) or on a 12-lead ECG, characterized by the absence of discrete P waves, replacement by fibrillatory waves of variable amplitude, morphology, and cycle length, and completely irregular RR intervals. NOAF was defined as the first occurrence of AF after admission in patients without a prior history of AF (11). The infarct-related artery (IRA) was determined by coronary angiography (CAG).

### Statistical analysis

Statistical analyses were performed using SPSS (version 27.0; Chicago, USA) and R (version 4.3.1; R Foundation for Statistical Computing). The Kolmogorov–Smirnov test was used to assess normality. Normally distributed continuous variables are presented as mean ± standard deviation (SD) and compared using Student's *t* test. Non-normally distributed continuous variables are presented as median (Q25, Q75) and compared using the Mann–Whitney *U* test. Variables with (*P* < 0.05) in univariable analyses or deemed clinically relevant for NOAF were entered into a multivariable logistic regression model using stepwise forward method. Restricted cubic splines (RCS) were used to explore the dose–response relationship between IBI and NOAF. Receiver operating characteristic (ROC) curves, net reclassification improvement (NRI), and integrated discrimination improvement (IDI) were used to evaluate the incremental discriminative ability of IBI for NOAF. A two-sided (*P* < 0.05) was considered statistically significant.

## Results

### Baseline characteristics of the study population

A total of 696 patients were included; of these, 62 (8.9%) developed NOAF. Baseline characteristics are summarized in Table 1. Compared with patients without NOAF, those with NOAF were older (71.13 ± 10.22 years vs. 63.38 ± 13.10 years, *P* < 0.001). The left ventricular ejection fraction (LVEF) was significantly lower in the NOAF group than in the non-NOAF group (47.65 ± 9.34% vs. 51.88 ± 6.62%, *P* < 0.001). Laboratory findings showed that the levels of NT-proBNP, neutrophil count, CRP, NLR, and IBI in the NOAF group were higher than those in the non-NOAF group (*P* < 0.005), while the level of lymphocyte count was lower (*P* < 0.001).

**Table 1:** Baseline characteristics of the study population.

Variables	Total (*n* = 696)	Non-NOAF (*n* = 634)	NOAF (*n* = 62)	*P*
Age, years	64.07 ± 13.05	63.38 ± 13.10	71.13 ± 10.22	<.001
Male, *n* (%)	501 (71.98)	462 (72.87)	39 (62.90)	0.095
BMI, kg/m^2^	24.60 ± 3.43	24.57 ± 3.32	24.85 ± 4.43	0.635
Heart rate, bpm	79.44 ± 13.97	79.33 ± 13.88	80.52 ± 14.92	0.525
SBP, mmHg	126.50 ± 21.72	126.61 ± 21.21	125.31 ± 26.60	0.709
DBP, mmHg	78.59 ± 13.71	78.73 ± 13.67	77.16 ± 14.19	0.390
WBC, 10*9 /L	10.10 ± 3.30	10.03 ± 3.31	10.80 ± 3.11	0.080
Neu, 10*9 /L	7.93 ± 3.92	7.82 ± 3.98	9.01 ± 3.07	0.023
Lym, 10*9 /L	1.68 ± 1.14	1.73 ± 1.17	1.26 ± 0.55	<.001
Hb, g/L	140.50 ± 16.60	140.47 ± 16.62	140.84 ± 16.60	0.866
PLT, 10*9 /L	214.84 ± 59.15	215.81 ± 59.20	204.94 ± 58.28	0.167
NLR	6.80 ± 6.03	6.58 ± 5.96	9.05 ± 6.31	0.002
IBI	34.42 ± 51.51	31.75 ± 48.80	61.69 ± 68.40	0.001
TG, mmol/L	4.41 ± 1.07	4.41 ± 1.06	4.36 ± 1.21	0.709
TC, mmol/L	1.44 ± 0.82	1.43 ± 0.81	1.52 ± 0.87	0.454
LDL-C, mmol/L	2.75 ± 0.91	2.76 ± 0.91	2.69 ± 0.97	0.567
HDL-C, mmol/L	1.06 ± 0.29	1.06 ± 0.29	1.02 ± 0.31	0.266
CRP, mg/L	4.09 (1.09, 6.96)	3.70 (1.02, 6.80)	5.78 (2.62, 9.60)	0.001
TnI, ng/ml	22.85 ± 16.02	22.70 ± 16.07	24.44 ± 15.49	0.415
NT-proBNP, pg/ml	2,058.0 (1,085.3, 4,294.0)	1,988.5 (1,064.8, 4,092.2)	3,484.3 (1,801.4, 5,479.4)	0.001
Hypertension, *n* (%)	284 (40.80)	254 (40.06)	30 (48.39)	0.203
Diabetes, *n* (%)	160 (22.99)	145 (22.87)	15 (24.19)	0.813
Stroke, *n* (%)	77 (11.06)	74 (11.67)	3 (4.84)	0.102
Pacemakers, *n* (%)	15 (2.16)	12 (1.89)	3 (4.84)	0.142
Smoking, *n* (%)	281 (40.37)	260 (41.01)	21 (33.87)	0.274
Aspirin, *n* (%)	643 (92.39)	583 (91.96)	60 (96.77)	0.265
P2Y12, n(%)	668 (95.98)	607 (95.74)	61 (98.39)	0.501
β-blockers, *n* (%)	563 (80.89)	509 (80.28)	54 (87.10)	0.193
Statins, *n* (%)	657 (94.40)	596 (94.01)	61 (98.39)	0.253
ACEI/ARB, *n* (%)	367 (52.73)	336 (53.00)	31 (50.00)	0.652
LVEF, %	51.50 ± 7.00	51.88 ± 6.62	47.65 ± 9.34	<.001
KILLIP class, *n* (%)				0.071
I	584 (83.91)	538 (84.86)	46 (74.19)	
II	46 (6.61)	41 (6.47)	5 (8.06)	
III	20 (2.87)	16 (2.52)	4 (6.45)	
IV	46 (6.61)	39 (6.15)	7 (11.29)	
LAD, *n*(%)	363 (52.16)	336 (53.00)	27 (43.55)	0.155
LCX, *n*(%)	80 (11.49)	74 (11.67)	6 (9.68)	0.638
RCA, *n* (%)	235 (33.76)	208 (32.81)	27 (43.55)	0.088

IBI, inflammatory burden index; TG, triglycerides; TC, total cholesterol; WBC, white blood cell; Neu, neutrophil; Lym, lymphocyte; PLT, platelet; Hb, hemoglobin; NLR, neutrophil-to-lymphocyte ratio; BMI, body mass index; LVEF, left ventricular ejection fraction; SBP, systolic blood pressure; DBP, diastolic blood pressure; ARB, angiotensin II receptor blocker; ACEI, angiotensin converting enzyme inhibitors; HDL-C, high-density leptin cholesterol; LDL-C, low-density leptin cholesterol; CRP, C-reactive protein; TnT, troponin I; NT-proBNP, N-terminal pro-B-type natriuretic peptide; LCX, left circumflex artery; LAD, left anterior descending artery; RCA, right coronary artery.

### Univariate and multivariate logistic regression analysis

**Figure 2:**
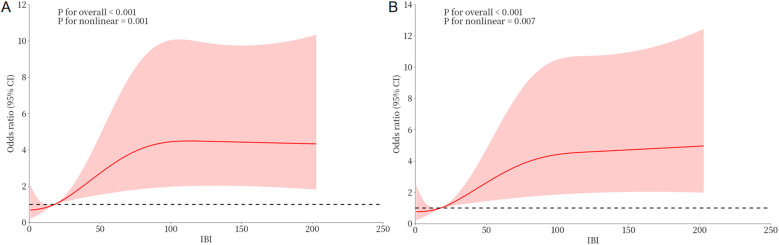
Dose-response relationship between IBI and NOAF from RCS analysis. **(A)** Unadjusted dose-response relationship between IBI and NOAF; **(B)** Adjusted dose-response relationship between IBI and NOAF. IBI, inflammatory burden index; NOAF, new-onset atrial fibrillation.

Univariable analyses were performed for all variables listed in Supplementary Table S1. Age, CRP, NT-proBNP, LVEF, Killip class >1, IBI, NLR, neutrophil count, and lymphocyte count were each significantly associated with NOAF (*P* < 0.05). Variables with (*P* < 0.05) in univariable analyses or of clinical relevance were entered into multivariable logistic regression. In the final model, LVEF (OR = 0.928, 95% CI: 0.895–0.962), age (OR = 1.048, 95% CI: 1.022–1.075), and IBI (OR = 1.007, 95% CI: 1.003–1.011) were independently associated with NOAF (Table 2). RCS analysis suggested a non-linear dose–response relationship between IBI and NOAF (Figure 2).

**Table 2:** Multivariate regression analysis for NOAF.

Variables	OR (95%CI)	*P*
IBI	1.007 (1.003–1.011)	<.001
LVEF, %	0.928 (0.895–0.962)	<.001
Age, years	1.048 (1.022–1.075)	<.001

IBI, inflammatory burden index; LVEF, left ventricular ejection fraction; NOAF, new-onset atrial fibrillation.

### Discriminatory accuracy and reclassification accuracy of IBI for NOAF

**Figure 3:**
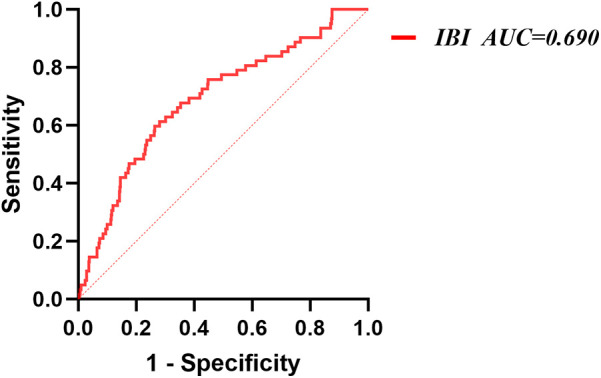
Receiver operating characteristic analysis (ROC) of IBI for NOAF. IBI, inflammatory burden index; NOAF, new-onset atrial fibrillation.

ROC analysis showed that IBI demonstrated moderate discriminatory ability for NOAF (AUC = 0.690, 95% CI: 0.620–0.761). The optimal cutoff was 36.85, yielding a sensitivity of 59.7% and a specificity of 73.7% (Figure 3).

**Figure 4:**
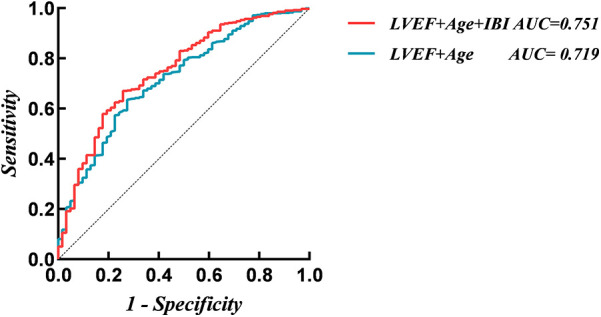
Receiver operating characteristic analysis (ROC) of models for new-onset atrial fibrillation. IBI, inflammatory burden index; LVEF, left ventricular ejection fraction.

A traditional model was constructed based on the independent risk factors identified by the multivariate analysis, including age, and LVEF. ROC analysis results showed that the AUC of the traditional model for predicting NOAF during hospitalization was 0.719. After adding IBI, the AUC increased to 0.751. DeLong's test indicated that this improvement was statistically significant (*Z* = 2.203, *P* = 0.028) (Figure 4). Adding IBI also significantly improved reclassification and overall discrimination (NRI = 0.617, 95% CI: 0.360–0.873, *P* < 0.01; IDI = 0.026, 95% CI: 0.007–0.046, *P* = 0.008) (Table 3).

**Table 3:** Incremental value of IBI for NOAF.

Models	NRI		IDI	
	Estimate (95% CI)	*P*	Estimate (95% CI)	*P*
LVEF + age	Reference	–	Reference	–
LVEF + age + IBI	0.617 (0.360–0.873)	<.001	0.026 (0.007–0.046)	0.008

IBI, inflammatory burden index; LVEF, left ventricular ejection fraction; NOAF, new-onset atrial fibrillation.

## Discussion

The main findings of this study were as follows. First, elevated IBI was an independent risk factor for NOAF in STEMI patients. Second, there was a non-linear dose-response relationship between the IBI and NOAF. Third, integration of IBI could improve the risk stratification for NOAF in STEMI patients.

Inflammation is a fundamental pathological process shared by STEMI and AF (5, 6). Previous studies have shown that NLR and CRP, as key indicators of cardiovascular risk, can effectively enhance the risk stratification assessment of patients with various cardiovascular diseases (12). Recently, the IBI, as an emerging inflammatory marker, has been widely used in the risk stratification of cancer patients (7–9). Compared with single inflammatory markers, the IBI, which is calculated from neutrophils, lymphocytes, and CRP through a specific formula, can provide a more stable and accurate assessment of inflammation, thus more precisely reflecting the body's inflammatory state and predicting the prognosis (8, 9, 13). In a cross-sectional study involving 15,325 American adults, Yu et al. found that the IBI level was independently associated with the prevalence of cardiovascular disease (CVD) and was closely related to the occurrence and development of CVD (7). In another multicenter study, the results indicated that the IBI was positively correlated with the risk of poor prognosis in patients with acute stroke (8). However, the relationship between IBI and NOAF after PCI in STEMI patients remains unclear. Our study found that in STEMI patients, elevated IBI was an independent risk factor for NOAF after PCI, and there was a non-linear dose-response relationship between them. CRP, as a traditional inflammatory marker, plays an important role in predicting the onset and prognosis of cardiovascular and cerebrovascular diseases (14, 15). Neutrophils and lymphocytes, as the two main types of peripheral inflammatory cells and important components of the immune system, play an indispensable role in the occurrence, development, and subsequent pathological processes of inflammation (16–18). During the acute phase of STEMI, the body is in a stress state. At this time, the elevated levels of catecholamines and cortisol lead to a decrease in lymphocyte count and an increase in neutrophil count and CRP level (19). A large number of inflammatory cells infiltrate the atrial tissue and release various cytokines and inflammatory mediators. These mediators can alter the function of ion channels, creating conditions for the occurrence of reentrant arrhythmias (20, 21). Additionally, the inflammatory state indicated by an elevated IBI may activate the sympathetic nervous system or inhibit the vagus nervous system (22, 23). Sympathetic nerve excitation can increase the automaticity of atrial myocytes and shorten the effective refractory period of the atria, making the atria more prone to fibrillation (24, 25). A decrease in vagal tone weakens its protective effect on the cardiac rhythm (26). The imbalance between the two provides a favorable neuroregulatory environment for the occurrence of AF (24, 26). The IBI analyzes the balance between acute inflammation and immune-mediated inflammation by comprehensively considering CRP and NLR, thus presenting a relatively complete picture of the body's pro-inflammatory and immune states. This may, to some extent, explain the relevant results of our study.

In addition, the results of our study are consistent with previous research findings (27, 28), further confirming that age and LVEF are also independent risk factors for NOAF in patients with STEMI. After incorporating the IBI into the traditional risk model, the predictive ability of the model for NOAF was significantly improved. In STEMI complicated with NOAF, accurate identification of risk factors is crucial for early intervention and improving prognosis. Incorporating IBI into the category of risk factors for NOAF helps clinicians more comprehensively and accurately assess the risk of NOAF after PCI in STEMI patients, providing additional information for the formulation of individualized prevention and treatment strategies. In clinical practice, for STEMI patients with high IBI, low LVEF, and advanced age, extended rhythm monitoring may be considered. In our study, the IBI cut-off was 36.85. To enhance clinical practicality, a more pragmatic stratification approach could be adopted in practice (e.g., using rounded thresholds such as >35 or >40) to guide enhanced patient monitoring. Moreover, it is important to emphasize that this cut-off requires external validation in independent cohorts to assess its generalizability and robustness.

### Limitations

This study has several limitations that need to be addressed. First, it is a single-center retrospective study, and the generalization of its conclusions may require validation through more multicenter randomized controlled trials. Second, in our study, other comorbidities or acute conditions may influence inflammatory markers and increase the risk of NOAF. As this is a retrospective study, although we controlled for several factors, residual confounding may persist and could introduce potential bias. Third, although the results of this study confirm the association between the IBI and NOAF in patients with STEMI, the specific underlying mechanism may need further basic research for clarification. Fourth, due to the lack of long-term electrocardiogram monitoring of patients before admission, some previous histories of atrial fibrillation, especially cryptogenic atrial fibrillation, may have been overlooked. Fifth, NOAF during the chronic phase after discharge following STEMI is also an important topic that merits attention. However, we currently lack follow-up data in this area, and more targeted future studies are warranted.

## Conclusions

Elevated IBI is an independent risk factor for NOAF after PCI in STEMI patients. Integration of IBI can improve the risk stratification for NOAF in STEMI patients. IBI may serve as a simple tool to identify high-risk patients, thereby supporting the implementation of intensified monitoring strategies and individualized management in this population.

## Data Availability

The raw data supporting the conclusions of this article will be made available by the authors, without undue reservation.
