# Supplementary material for: Predictive value of inflammatory burden index for new-onset atrial fibrillation in STEMI patients
Source: Front Cardiovasc Med. 2025 Sep 16;12:1599152. doi: 10.3389/fcvm.2025.1599152 (PMC12479414; doi:10.3389/fcvm.2025.1599152)
Supplement: Supplementary file 1 [file Table1.pdf]

Table S1. Univariate Regression Analysis for NOAF

Variables	β	S.E	*Z*	OR (95%CI)	*P*
Male, n (%)	0.46	0.28	1.66	1.58 (0.92 ~ 2.73)	0.098
Hypertension, n (%)	0.34	0.27	1.27	1.40 (0.83 ~ 2.37)	0.205
Diabetes mellitus, n (%)	0.07	0.31	0.24	1.08 (0.58 ~ 1.98)	0.813
Stroke, n (%)	-0.96	0.60	-1.58	0.38 (0.12 ~ 1.26)	0.114
Smoking, n (%)	-0.31	0.28	-1.09	0.74 (0.43 ~ 1.28)	0.276
Aspirin, n (%)	0.96	0.73	1.32	2.62 (0.62 ~ 11.04)	0.188
P2Y12, n(%)	1.00	1.03	0.97	2.71 (0.36 ~ 20.29)	0.331
β-blockers, n (%)	0.51	0.39	1.29	1.66 (0.77 ~ 3.57)	0.197
Statins, n (%)	1.36	1.02	1.33	3.89 (0.52 ~ 28.82)	0.184
ACEI/ARB, n (%)	-0.12	0.27	-0.45	0.89 (0.53 ~ 1.49)	0.652
KILLIP class > 1, n (%)	0.67	0.31	2.15	1.95 (1.06 ~ 3.58)	0.032
LAD, n(%)	-0.38	0.27	-1.42	0.68 (0.40 ~ 1.16)	0.157
LCX, n(%)	-0.21	0.45	-0.47	0.81 (0.34 ~ 1.95)	0.639
RCA, n(%)	0.46	0.27	1.70	1.58 (0.93 ~ 2.68)	0.090
IBI	0.01	0.00	3.83	1.01 (1.01 ~ 1.01)	<.001
WBC ,10*9/L	0.07	0.04	1.75	1.07 (0.99 ~ 1.15)	0.081
Neu, 10*9/L	0.05	0.03	1.97	1.05 (1.01 ~ 1.11)	0.049
Lym, 10*9/L	-0.71	0.22	-3.26	0.49 (0.32 ~ 0.75)	0.001
Hb, g/L	0.00	0.01	0.17	1.00 (0.99 ~ 1.02)	0.866
PLT ,10*9/L	-0.00	0.00	-1.38	1.00 (0.99 ~ 1.00)	0.167
NLR	0.05	0.02	2.90	1.05 (1.02 ~ 1.08)	0.004
Age, years	0.05	0.01	4.35	1.06 (1.03 ~ 1.08)	<.001
BMI, kg/m^2	0.02	0.04	0.60	1.02 (0.95 ~ 1.10)	0.546
Heart rate, bpm	0.01	0.01	0.64	1.01 (0.99 ~ 1.02)	0.525
SBP, mmHg	-0.00	0.01	-0.45	1.00 (0.99 ~ 1.01)	0.651
DBP, mmHg	-0.01	0.01	-0.86	0.99 (0.97 ~ 1.01)	0.390
CRP, mg/L	0.10	0.03	3.22	1.11 (1.04 ~ 1.18)	0.001
TnI, ng/mL	0.01	0.01	0.67	1.01 (0.99 ~ 1.02)	0.415
NT-proBNP, pg/mL	0.84	0.29	2.89	2.31 (1.31 ~ 4.07)	0.004
Triglycerides, mmol/L	-0.05	0.13	-0.37	0.95 (0.74 ~ 1.22)	0.709
Total cholesterol, mmol/L	0.11	0.15	0.75	1.12 (0.83 ~ 1.51)	0.454
LDL-C, mmol/L	-0.09	0.15	-0.57	0.92 (0.68 ~ 1.23)	0.567
HDL-C, mmol/L	-0.62	0.55	-1.12	0.54 (0.18 ~ 1.59)	0.261
LVEF, %	-0.08	0.02	-4.44	0.93 (0.89 ~ 0.96)	<.001

IBI = inflammatory burden index; WBC = white blood cell; Neu = neutrophil; Lym = lymphocyte; PLT = platelet; Hb = Hemoglobin; NLR = neutrophil-to-lymphocyte ratio; BMI = body mass index; LVEF = left ventricular ejection fraction; SBP = systolic blood pressure; DBP = diastolic blood pressure; ARB = angiotensin II receptor blocker; ACEI = angiotensin converting enzyme inhibitors; HDL-C = high-density leptin cholesterol; LDL-C = low-density leptin cholesterol; hs-CRP = high sensitivity C-reactive protein; TnI = troponin I; NT-proBNP = N-terminal pro-B-type natriuretic peptide; LCX = left circumflex artery; LAD = left anterior descending artery; RCA = right coronary artery.
